# Fully automated pelvic bone segmentation in multiparameteric MRI using a 3D convolutional neural network

**DOI:** 10.1186/s13244-021-01044-z

**Published:** 2021-07-07

**Authors:** Xiang Liu, Chao Han, He Wang, Jingyun Wu, Yingpu Cui, Xiaodong Zhang, Xiaoying Wang

**Affiliations:** Department of Radiology, Peking University First Hospital, No.8 Xishiku Street, Xicheng District, Beijing, 100034 China

**Keywords:** Pelvic bones, Segmentation, Multiparametric magnetic resonance imaging, Convolutional neural network, Deep learning

## Abstract

**Background:**

Accurate segmentation of pelvic bones is an initial step to achieve accurate detection and localisation of pelvic bone metastases. This study presents a deep learning-based approach for automated segmentation of normal pelvic bony structures in multiparametric magnetic resonance imaging (mpMRI) using a 3D convolutional neural network (CNN).

**Methods:**

This retrospective study included 264 pelvic mpMRI data obtained between 2018 and 2019. The manual annotations of pelvic bony structures (which included lumbar vertebra, sacrococcyx, ilium, acetabulum, femoral head, femoral neck, ischium, and pubis) on diffusion-weighted imaging (DWI) and apparent diffusion coefficient (ADC) images were used to create reference standards. A 3D U-Net CNN was employed for automatic pelvic bone segmentation. Additionally, 60 mpMRI data from 2020 were included and used to evaluate the model externally.

**Results:**

The CNN achieved a high Dice similarity coefficient (DSC) average in both testing (0.80 [DWI images] and 0.85 [ADC images]) and external (0.79 [DWI images] and 0.84 [ADC images]) validation sets. Pelvic bone volumes measured with manual and CNN-predicted segmentations were highly correlated (*R*^2^ value of 0.84–0.97) and in close agreement (mean bias of 2.6–4.5 cm^3^). A SCORE system was designed to qualitatively evaluate the model for which both testing and external validation sets achieved high scores in terms of both qualitative evaluation and concordance between two readers (ICC = 0.904; 95% confidence interval: 0.871–0.929).

**Conclusions:**

A deep learning-based method can achieve automated pelvic bone segmentation on DWI and ADC images with suitable quantitative and qualitative performance.

**Supplementary Information:**

The online version contains supplementary material available at 10.1186/s13244-021-01044-z.

## Keypoints

- 3D U-Net exhibits good performance for segmentation of normal pelvic bones.
- A SCORE system was designed for the qualitative evaluation of segmentation.
- It lays a foundation for the detection of pelvic bony metastases.

## Background

Multiparametric magnetic resonance imaging (mpMRI) has previously demonstrated high sensitivity and specificity in diagnosing pelvic bone metastases [1]. As a well-recognised and widely used sequence in oncologic imaging, diffusion-weighted imaging (DWI) has been reported to be able to offer both qualitative (signal intensity) and quantitative (apparent diffusion coefficient [ADC] maps) information for lesion detection and characterisation [2–4]. Adverse bone events, such as pathological fracture and spinal cord compression, were often led by bone metastases [5, 6]. Therefore, timely and accurate detection of bone metastases on DWI and ADC images is of great significance in guiding patient care and assessing therapeutic benefits.

When radiologists interpret pelvic magnetic resonance imaging (MRI) images, bone metastasis location should first be determined, followed by size and ADC measurement of the metastatic foci. Thus, the initial step to achieve accurate bone metastases detection on DWI and ADC images requires accurate skeleton segmentation with their semantic labels. It is the first step in developing an automated method for quantifying skeletal metastatic tumour burden. Deep learning techniques lead the transformation of manual segmentation into automated segmentation [7–9]. For example, fully convolutional neural networks (CNNs) such as the U-Net model proposed by Ronneberger et al. [10] and the V-Net model proposed by Milletari et al. [11] have significantly increased the potential of automated image analysis to an unprecedented level.

Previously, several studies have reported CNNs for segmentation of normal bone structures on CT images and bone scans [12, 13], however, only a few studies of automatic segmentation of normal bone structures on MR images are available. Quantitative evaluation is a routine method for segmentation models, while qualitative evaluation has a better correlation with clinical practice [14]. Quantitative and qualitative evaluations are complementary, and together evaluate the segmentation model more completely [15]. The purpose of this study is to develop a 3D U-Net model to automatically segment different pelvic bony structures on DWI and ADC images—lumbar vertebra, sacrococcyx, ilium, acetabulum, femoral head, femoral neck, ischium, and pubis, which represent the most frequent sites of bone metastases for prostate cancer [16], and test its feasibility quantitively and qualitatively. This research may provide essential localisation information for subsequent research on pelvic bone metastases.

## Materials and methods

This retrospective study was performed with permission from the local Institutional Ethical Committee. The need for written informed consent was waived.

### Patients enrollment

**Fig. 1:**
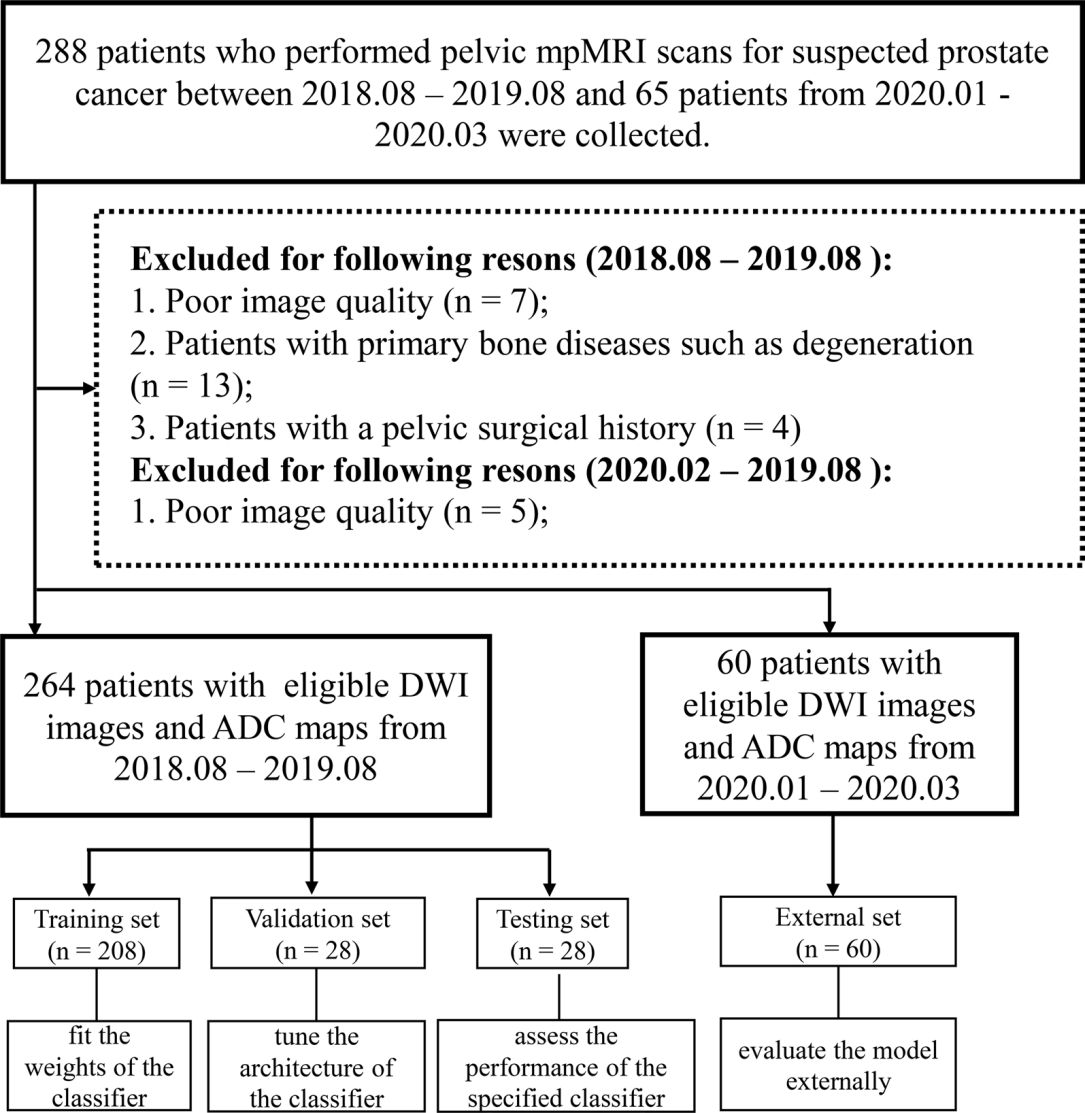
The workflow of patient enrollment and distribution

For algorithm development, we retrospectively collected 288 patients who performed pelvic mpMRI scans for suspected prostate cancer at our institution between August 2018 and August 2019. The inclusion criteria included the following: (1) no sign of obvious bone structure abnormality, (2) DWI images with low (0 s/mm^2^) and high (800 or 1000 s/mm^2^) b values and ADC maps accordingly reconstructed, and (3) good image quality without obvious artefacts. The exclusion criteria included those with: (1) pelvic surgical history, and (2) bone diseases that occurred within the pelvis such as primary bone tumour and necrosis. In total, 264 patients remained enrolled for this study, with patients excluded for various reasons including poor image quality (*n* = 7), those with primary bone diseases such as degeneration, hemangioma and sarcoma (*n* = 13), and those with a pelvic surgical history (*n* = 4). Retrospectively, a further 60 consecutive pelvic mpMRI data were collected between January 2020 and March 2020 from our institution that satisfied the above inclusion criteria in order to provide external validation (5 patients were excluded due to poor image quality) (Fig. 1). All mpMRI data were de-identified before inclusion.

### mpMRI data

A consecutive cohort of pelvic mpMRI data from our hospital was retrospectively collected to develop and test the algorithm used in this study. Details of the imaging parameters and techniques of the DWI sequence are summarised in Table 1. The ADC maps were calculated from two *b* values (0, 800 or 1000 s/mm^2^) using the corresponding scanner software.

**Table 1:** Imaging system and typical parameters

MR Vendor	Algorithm Development^#^ (*N* = 264)			External Set^*^ (*N* = 60)	Typical parameters (DWI sequence)
	Training set (*N* = 208)	Validation set (*N* = 28)	Testing set (*N* = 28)		
3.0 T Discovery (Ge healthcare, Milwaukee, WI)	*N* = 157	*N* = 20	*N* = 20	*N* = 41	B value: 0, 800 s/mm^2^; Imaging matrix:256 × 256; Echo time: 60 ms; Repetition time: 3000 ms; Field of view: 360 mm; Section thickness: 4 mm Number of slices: 25
3.0 T Intera (Philips Medical Systems, Best, the Netherlands)	*N* = 37	*N* = 5	*N* = 5	*N* = 11	B value: 0, 1000 s/mm^2^; Imaging matrix: 240 × 240; Echo time:78 ms; Repetition time: 4959 ms; Field of view: 360 mm; Section thickness:7 mm Number of slices: 28
1.5 T Avanto (Siemens Medical Solutions, Erlangen, Germany)	*N* = 14	*N* = 3	*N* = 3	*N* = 8	B value: 0, 800 s/mm^2^; Imaging matrix: 156 × 180; Echo time:54 ms; Repetition time: 3300 ms; Field of view: 360 mm; Section thickness: 7 mm Number of slices: 24

^#^The data in algorithm development were collected between August 2018 and August 2019

^*^The data in external set were collected between January 2020 and March 2020

### Algorithm development

The neural network model developed for the segmentation of pelvic bone structures on axial DWI and ADC images is a 3D U-Net [17], which replaced all the 2D operations of U-Net architecture with 3D counterparts (Additional file 1). Considering the 3D nature of bones, pelvic bone segmentation may be better performed using 3D U-Net, where 3D segmentation can utilise the inherent dependency between the spatial location of multiple slices [18] in contrast to 2D CNN which ignores 3D continuity of segmented bones between slices.

The developed CNN takes the combination of 264 DWI images (*b* = 800 or 1000 s/mm^2^) and 264 ADC images acquired from three vendors as input, and each image sequence was an independent input data. The CNN model was developed with one input channel. The 264 patients were randomly divided into either the training (*n* = 208), validation (*n* = 28) or testing (*n* = 28) sets with a ratio of 8:1:1. The independent dataset in the external validation set (*n* = 60) was used to evaluate further the performance and generalizability of the 3D U-Net model.

The training set was used to fit the classifier weights, and the validation set was used to tune the classifier architecture. The testing set was used to assess the fully specified classifier performance, and the external set was used to externally evaluate the model using data collected from various times (Fig. 1).

The original sizes of DWI images (acquired from three vendors) were 24 × 180 × 156 (*z*, *y*, *x*); 25 × 256 × 256; 24 × 224 × 224. All the input images were unified and resized to 64 × 256 × 256 (*z*, *y*, *x*) before training in order to maintain the optimal image features. All of the images were normalised of pixel intensity to [0, 1]. The CNN was coded by Python3.6, Pytorch 0.4.1, Opencv, Numpy, SimpleITK, and trained on the GPU NVIDIA Tesla P100 16G. A total of 300 epochs of training were performed. The Adam Optimizer was employed to minimise loss with a learning rate of 0.0001, a batch of size 2, and a binary cross-entropy loss function. Other hyperparameters tuning (such as weight initialisation and dropout for regularisation) were randomly searched and automatically executed in the validation set during U-Net development.

### Manual annotation

**Fig. 2:**
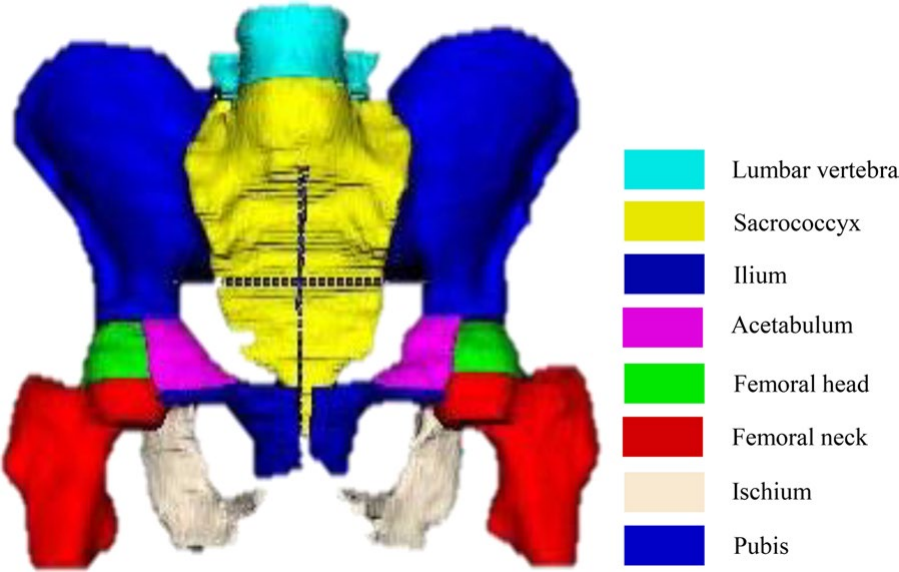
Reconstruction of manual segmentations of pelvic bones. A multiclass label was created for the patients; 1 = lumbar vertebra, 2 = sacrococcyx, 3 = ilium, 4 = acetabulum, 5 = femoral head, 6 = femoral neck, 7 = ischium, 8 = pubis

Under the supervision of a board-certified radiology expert (with more than 15 years of reading experience), a radiology resident with three years of reading experience evaluated all mpMRI examinations and, section by section, manually labelled the pelvic bones (containing bone marrow and cortex) on DWI images with high b values. The labels were created with the following values: 1 = lumbar vertebra, 2 = sacrococcyx, 3 = ilium, 4 = acetabulum, 5 = femoral head, 6 = femoral neck, 7 = ischium, and 8 = pubis. A connected domain (CD) is defined as the part of a label area with a continuous structure in 3D space, and one label may contain multiple CDs. As shown in Fig. 2, a lumbar vertebral label is a single CD, while the ilium label contains two CDs (one on the left, and one on the right). Therefore, there are 14 CDs in the complete reconstructed pelvic bone, including one lumbar vertebra, one sacrococcyx, two ilia, two acetabula, two femoral head, two femoral necks, two ischia and two pubes.

Since DWI and ADC images were co-registered by the scanner (ADC maps were calculated from DWI images), the manually segmented labels on DWI images could be matched to the ADC maps. The supervisor reviewed all labels on ADC images that copied from DWI images and made corrections wherever necessary. Images were manually annotated with an image segmentation software (ITK-SNAP 3.6; Penn Image Computing and Science Laboratory, Philadelphia, Pa).

### Quantitative evaluation

The Dice Similarity Coefficient (DSC) between CNN-predicted and manual segmentation was computed to evaluate the segmentation accuracy of DWI images and ADC maps quantitatively. The DSC was defined as the volume of overlap between the CNN-predicted and manual segmentation, given by:

$${\text{DSC}} = \frac{{2 \times (P \cap M)}}{{P + M}}$$

where *P* and *M* are the volume of voxels in the predicted segmentation and manual annotation, respectively, and *P* ∩ *M* is the volume of voxels that are consistent between the two methods. The DSC ranged between 0 and 1, with higher values indicating a better segmentation performance.

The volume calculation also quantified the accuracy of bone segmentation. The Bland–Altman method was used to assess the volume difference between CNN-predicted and manual segmentations.

### Qualitative evaluation

**Fig. 3:**
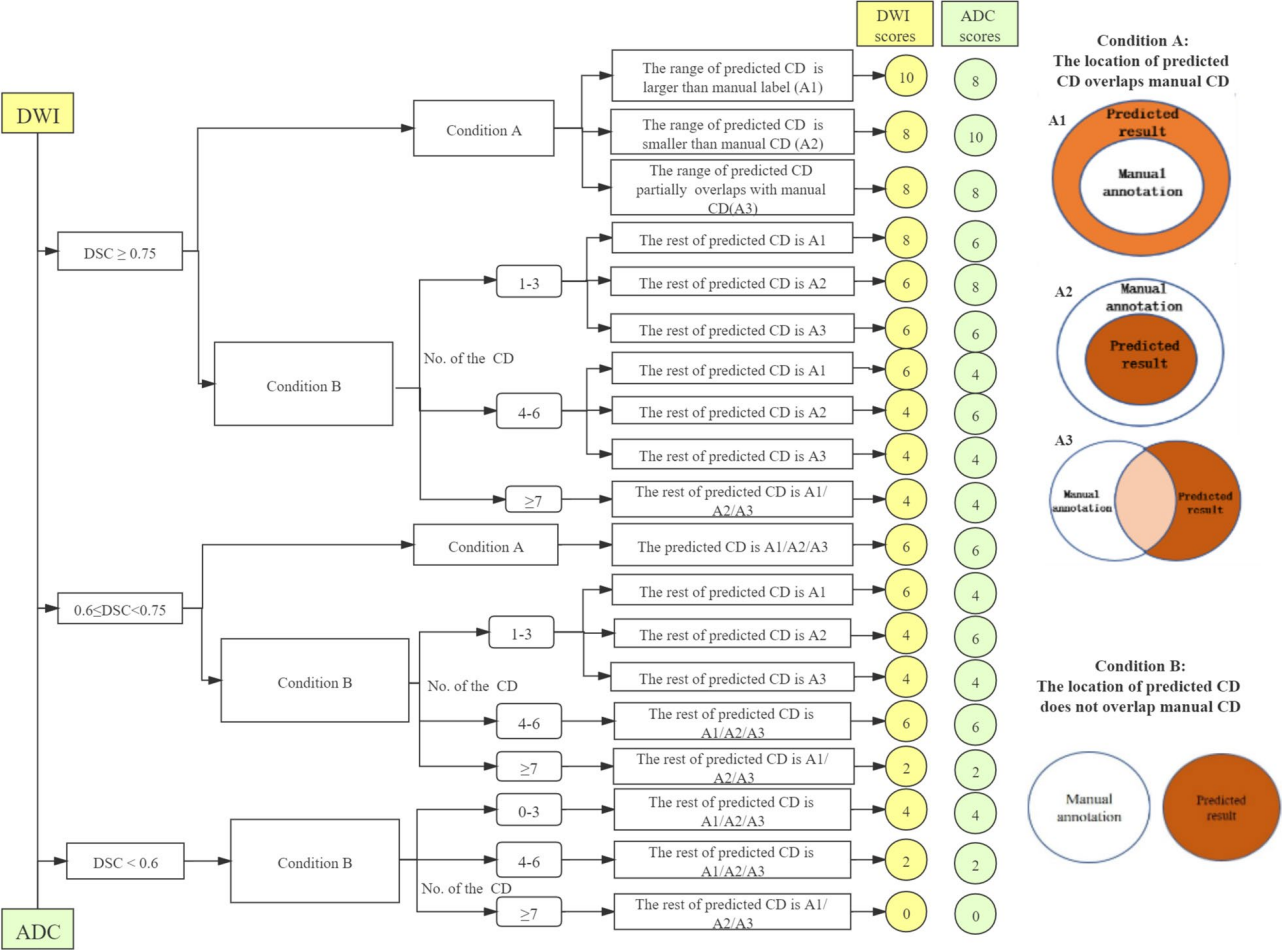
The SCORE system and evaluation criteria on DWI images and ADC maps. According to the diagram of the SCORE system, the evaluation of the predicted segmentations on DWI images and ADC maps included three steps: firstly to calculate the DSC value of each label predicted by the model, then to judge whether the location of the predicted CD of the label overlaps the manually segmented CD, and finally to calculate the average scores of the labels according to the number of the predicted CDs that do not overlap with manual CD. Condition A means that the location of the predicted CD overlaps manual CD, and the range of the predicted CD is larger than (A1) or less than (A2) the manual CD, or partially overlaps with the manual CD (A3). *CD* connected domain, *DSC* dice similarity coefficient

A qualitative SCORE system established in this work was used to evaluate the CNN-predicted results at the CD level, which focuses on evaluating whether the predicted results meet the requirements for clinical application. The evaluation criteria of DWI images and ADC maps at the CD level are shown in Fig. 3. Given the different usage of DWI and ADC images in evaluating bone metastases (i.e. DWI images for the qualitative diagnosis, ADC maps for quantitative measurement) [2, 3], the evaluation criteria are slightly different. For example, if the location of the predicted CD overlaps the manually annotated CD (i.e. Condition A), and the range of the predicted CD is larger than the manual CD (A1), which is clinically acceptable segmentation on DWI images for which does not affect the detection and localisation of lesions. While if the range of the predicted CD is smaller than the manual CD (A2), that is clinically acceptable for ADC images, eliminating the interference of other tissues to ADC measurements.

The score of a single CD is between 0–10, where CDs with the same label were used to calculate the average label score, and the score at the patient level is the average value of all labels. A radiology expert (with more than 15 years of reading experience) assessed the predicted results according to the SCORE system. A resident (with three years of reading experience) also qualitatively evaluated the segmentation performance of the model to compare the consistency between readers.

### Statistical analysis

One-way analysis of variance was performed to compare age and prostate-specific antigen (PSA) level (total PSA [T-PSA], free PSA [F-PSA], F/T-PSA) among four data sets (the training set, validation set, testing set and external validation set). A Kruskal–Wallis test was applied for the comparisons of these metrics among different vendors. Comparisons of DSCs between testing and external validation sets were performed using the Student’s t-test. One-way analysis of variance was applied to compare DSC values among different vendors, and the least significant difference (LSD) was used for post hoc multiple comparisons. To compare manual versus CNN-predicted bone volume, linear regression and Bland–Altman analyses were performed on both testing and external validation sets. The Student’s *t*-test was also used to compare the mean scores between DWI and ADC images. SCORE results between two readers were assessed using the single-measure intraclass correlation coefficient (ICC), with ICC > 0.75 considered an excellent concordance. A two-sided *p* < 0.05 was considered statistically significant.

## Results

### Patient demographics

The demographics of patients are presented in Table 2 and Additional file 2. There was no significant difference in age and PSA level (T-PSA, F-PSA, F/T-PSA) among the four data sets (Table 2, *p* > 0.05) and the three vendors (Additional file 2: Table S1, *p* > 0.05).

**Table 2:** Demographics of patients among different dataset

Characteristic	Algorithm development^#^ (N = 264)			External Set* (*N* = 60)	*F* Value	*P* value
	Training set	Validation set	Testing set			
No. of patients	208	28	28	60	–	–
Age (mean, years) (SD)	67.13 (9.75)	67.03 (9.93)	67.21 (9.35)	65.38 (13.07)	0.458	0.712
PSA (median, ng/ml)						
T-PSA (range)	10.34 (0.15, 156.00)	9.01 (0.77, 128.3)	8.96 (0.82, 27.99)	8.14 (1.38, 50.00)	1.349	0.259
F-PSA (range)	1.32 (0.09, 23.46)	1.08 (0.46, 9.05)	1.32 (0.34, 3.53)	1.43 (0.33, 13.20)	0.342	0.795
F/T-PSA (range)	0.13 (0.02, 0.36)	0.13 (0.05, 0.26)	0.16 (0.05, 0.24)	0.14 (0.05, 0.65)	2.077	0.104

*SD* standard deviation, *PSA* prostate specific antigen, *T-PSA* total PSA, *F-PSA* free PSA

^#^The data in algorithm development were collected between August 2018 and August 2019

^*^The data in external validation were collected between January 2020 and March 2020

**Fig. 4:**
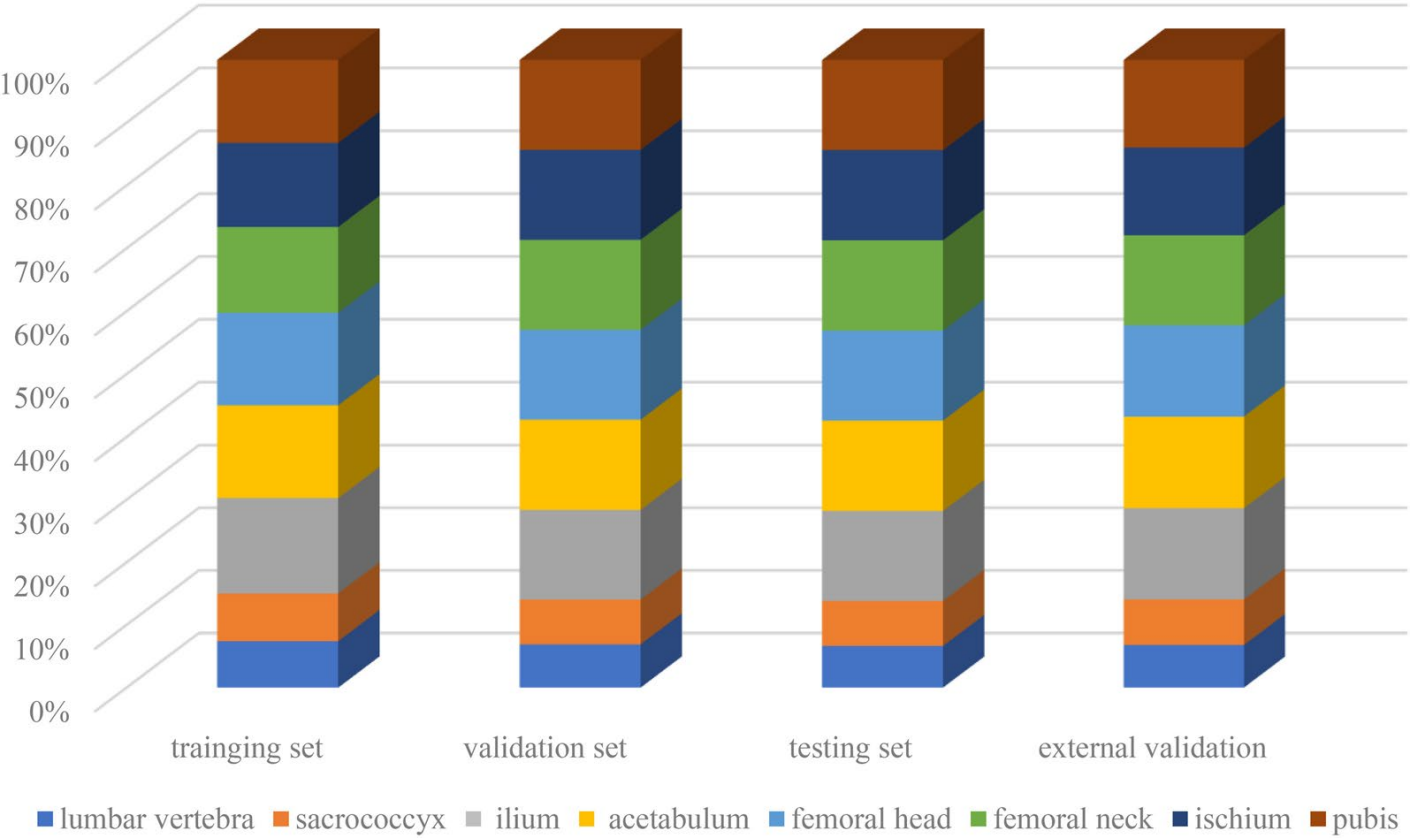
The distribution of different connected domains. The distribution of the connected domains among four data sets shows that all bones had roughly equal distribution in different data sets

The different field-of-views among different MR scanners may result in an unequal number of labels and CDs in each dataset, where some patients may lack the section of pubis or lumbar vertebra. The distribution of different CDs among the datasets can be seen in Fig. 4, where it shows that all CDs of the bones had roughly equal distribution among the four datasets, confirming the network’s results are not biased.

### Segmentation accuracy of pelvic bones

DWI and ADC images were independently inputted to train the model. Also, both the DWI and ADC were predicted independently to get their segmentation. As shown in Table 3, the model achieved good segmentation accuracy of pelvic bones in both testing and external validation sets, attaining average DSC values of 0.80 ± 0.05 and 0.79 ± 0.06 on DWI images and 0.85 ± 0.04 and 0.84 ± 0.04 on ADC images. The average DSC values from ADC images were higher than those of DWI images (testing set: *t*_DWI vs ADC_ = − 4.238, *P*_DWI vs ADC_ < 0.001; external validation set: *t*_DWI vs ADC_ = − 5.490, *P*_DWI vs ADC_ < 0.001).

**Table 3:** The DSC values of 3D U-Net in pelvic bones segmentation on DWI and ADC images

Pelvic bones	DWI images				ADC images			
	Testing Set^#^ (*N* = 28)	External Set^*^ (*N* = 60)	*t* value	*p* value	Testing set (*N* = 28)	External set (*N* = 60)	*t* value	*p* value
Lumbar vertebra	0.83 ± 0.06	0.81 ± 0.10	1.220	0.226	0.87 ± 0.05	0.83 ± 0.07	2.564	0.012
Sacrococcyx	0.84 ± 0.04	0.84 ± 0.07	1.193	0.236	0.86 ± 0.04	0.85 ± 0.05	0.667	0.507
Ilium	0.82 ± 0.06	0.80 ± 0.10	1.275	0.206	0.87 ± 0.04	0.86 ± 0.03	0.549	0.584
Acetabulum	0.77 ± 0.07	0.77 ± 0.08	-0.007	0.994	0.82 ± 0.08	0.82 ± 0.08	0.256	0.799
Femoral head	0.85 ± 0.06	0.84 ± 0.07	0.841	0.403	0.89 ± 0.06	0.87 ± 0.06	1.437	0.154
Femoral neck	0.84 ± 0.06	0.82 ± 0.08	1.402	0.165	0.89 ± 0.04	0.87 ± 0.08	1.660	0.101
Ischium	0.81 ± 0.07	0.77 ± 0.10	1.819	0.072	0.85 ± 0.07	0.84 ± 0.06	0.894	0.374
Pubis	0.68 ± 0.08	0.65 ± 0.11	0.922	0.359	0.77 ± 0.06	0.76 ± 0.08	0.694	0.490
Average^$^	0.80 ± 0.05	0.79 ± 0.06	1.452	0.150	0.85 ± 0.04	0.84 ± 0.04	1.788	0.077

Unless otherwise specified, data are mean DSC value ± standard deviations. DSC: Dice similarity coefficient

^#^The data in training set were collected between August 2018 and August 2019

^*^The data in external set were collected between January 2020 and March 2020

^$^Average indicates the mean value of all the pelvic bones

Additionally, the DSC values among different vendors on external validation sets for DWI and ADC images were compared. As shown in Table 4, the images from vendor 1 (3.0 T Discovery) attained significantly higher DSC averages than vendor 2 (1.5 T Intera) and vendor 3 (1.5 T Avanto) (DWI images: *F*_V1 vs V2 vs V3_ = 22.405, *P*_V1 vs V2 vs V3_ < 0.001; ADC images: *F*_V1 vs V2 vs V3_ = 13.420, *P*_V1 vs V2 vs V3_ < 0.001).

**Table 4:** The DSC values of 3D U-Net in pelvic bones segmentation on external validation set grouped by vendors

Pelvic bones	DWI images					ADC images				
	V1^#^ (*n* = 41)	V2^$^ (*n* = 11)	V3^&^ (*n* = 8)	*F* value	*p* value	V1^#^ (*n* = 41)	V2^$^ (*n* = 11)	V3^&^ (*n* = 8)	*F* value	*p* value
Lumbar vertebra	0.82 ± 0.10*	0.76 ± 0.13	0.76 ± 0.06	2.307	0.109	0.85 ± 0.07	0.80 ± 0.06	0.80 ± 0.08	2.756	0.073
Sacrococcyx	0.84 ± 0.05*	0.81 ± 0.05*	0.73 ± 0.11	12.582	0.000	0.87 ± 0.04*	0.83 ± 0.05	0.83 ± 0.05	4.111	0.021
Ilium	0.82 ± 0.04*	0.78 ± 0.03	0.71 ± 0.07	27.470	0.000	0.87 ± 0.03*	0.87 ± 0.03*	0.81 ± 0.03	15.708	0.001
Acetabulum	0.80 ± 0.06*	0.71 ± 0.08	0.69 ± 0.08	13.876	0.000	0.84 ± 0.07*	0.82 ± 0.07*	0.70 ± 0.12	10.273	0.001
Femoral head	0.86 ± 0.06*	0.81 ± 0.06*	0.79 ± 0.10	4.053	0.023	0.88 ± 0.06*	0.88 ± 0.07*	0.82 ± 0.05	2.703	0.076
Femoral neck	0.84 ± 0.06*	0.82 ± 0.07*	0.74 ± 0.13	5.828	0.005	0.87 ± 0.08	0.88 ± 0.06	0.84 ± 0.03	0.870	0.425
Ischium	0.80 ± 0.10*	0.70 ± 0.08	0.70 ± 0.07	6.877	0.002	0.85 ± 0.06*	0.82 ± 0.04	0.78 ± 0.08	6.315	0.003
Pubis	0.69 ± 0.09*	0.61 ± 0.14	0.60 ± 0.11	6.212	0.004	0.77 ± 0.09*	0.78 ± 0.04*	0.67 ± 0.05	6.299	0.003
Average^$^	0.81 ± 0.04*	0.75 ± 0.05	0.71 ± 0.07	22.405	0.001	0.85 ± 0.04*	0.83 ± 0.03	0.78 ± 0.04	13.420	0.001

Unless otherwise specified, data are mean DSC value ± standard deviations. DSC: Dice similarity coefficient

^#^V1 indicates Vendor 1: 3.0 T Discovery; ^$^V2 indicates Vendor 2: 1.5 T Intera; ^&^V3 indicates Vendor 3: 1.5 T Avanto

^*^indicates a significantly high DSC value

**Fig. 5:**
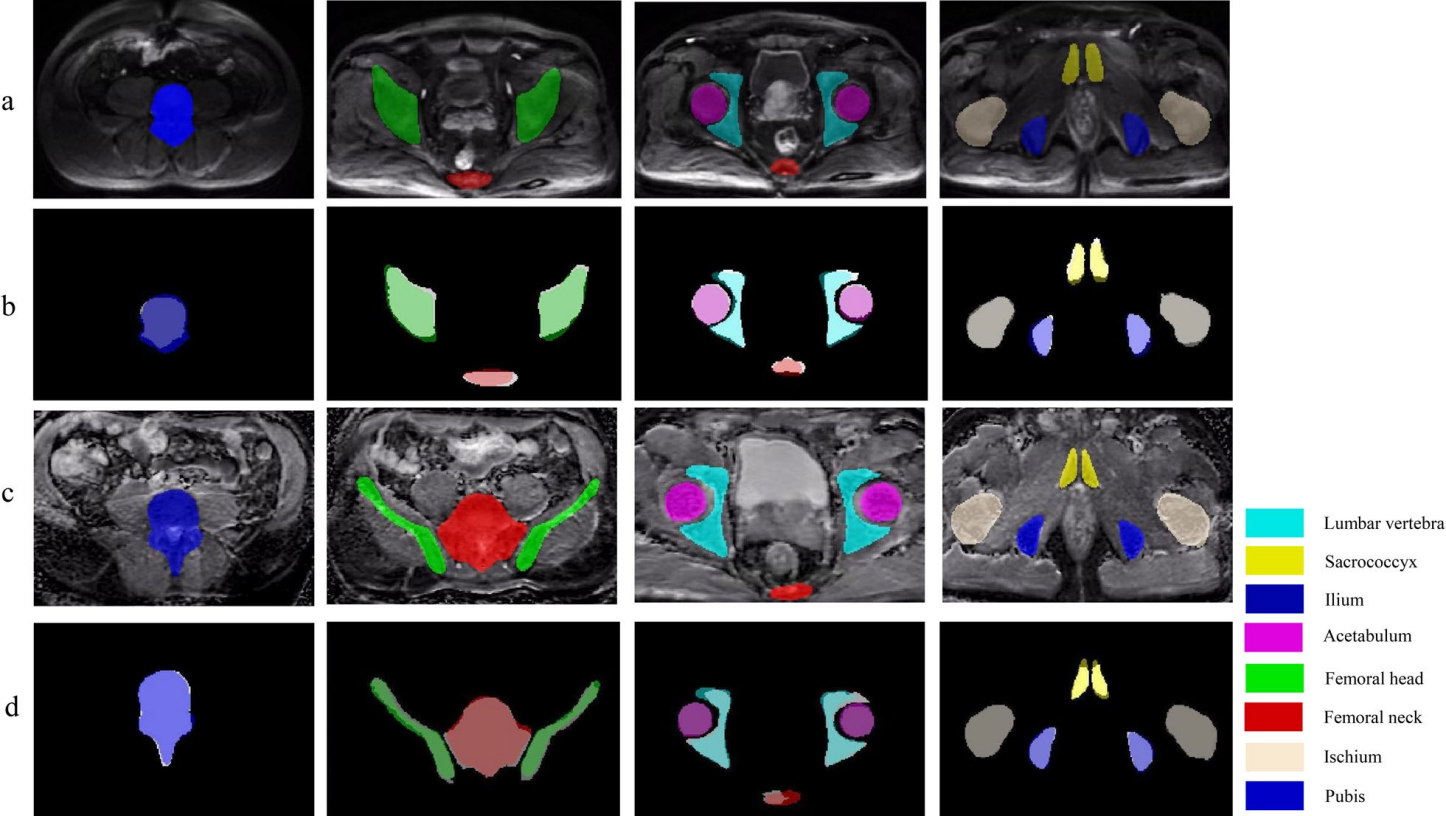
Examples of the comparison between CNN-predicted and manual segmentations. **a** Section examples of eight bones on DWI image; **b** The corresponding overlapping images between manual segmentation (white background) and CNN-predicted segmentation; **c** Section examples of eight bones on ADC image; **d** The corresponding overlapping images between manual segmentation (white background) and CNN-predicted segmentation

Figure 5 shows the predicted section examples of eight bony structures (lumbar vertebra, sacrococcyx, ilium, acetabulum, femoral head, femoral neck, ischium and pubis) from DWI and ADC images in the external validation set (Fig. 5a, c) and the corresponding overlapping images with manual segmentations (Fig. 5b, d).

### Volume calculation of pelvic bones

**Fig. 6:**
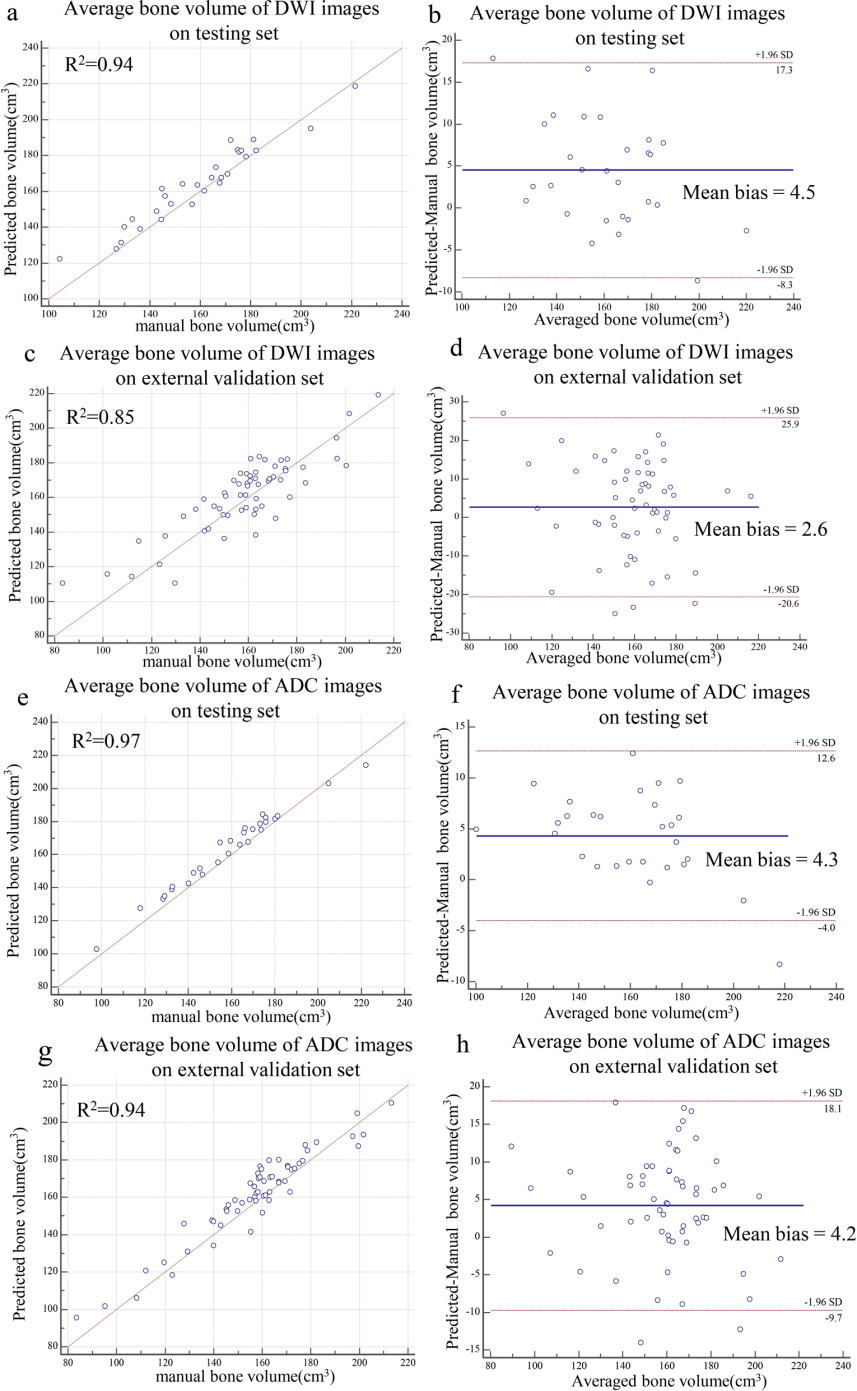
Agreement of bone volume assessments between CNN-predicted and manual segmentations. **a**, **c** Linear regression and (**b**, **d**) Bland–Altman analysis of bone volume assessments from DWI images. **e**, **g** Linear regression and (**f**, **h**) Bland–Altman analysis of bone volume estimates from ADC images

The overall bone volume between CNN-predicted and manual segmentation strongly correlated (DWI images in testing set: *R*^2^ = 0.94, Fig. 6a; DWI images in external validation set: *R*^2^ = 0.85, Fig. 6c; ADC images in testing set: *R*^2^ = 0.97, Fig. 6e; ADC images in external validation set: *R*^2^ = 0.94, Fig. 6g). When compared with the manual method, the automated CNN method slightly overestimated bone volume on both DWI images (testing set: mean bias = 4.5 cm^3^, 95% limits of agreement [LoA] were − 8.3 cm^3^ and 17.3 cm^3^, Fig. 6b; external validation set: mean bias = 2.6 cm^3^, 95% LoA were − 20.6 cm^3^ and 25.9 cm^3^, Fig. 6d) and ADC images (testing set: mean bias = 4.3 cm^3^, 95% LoA were − 4.0 cm^3^ and 12.6 cm^3^, Fig. 6f; external validation set: mean bias = 4.2 cm^3^, 95% LoA were − 9.7 cm^3^ and 18.1 cm^3^, Fig. 6h). The detailed volume difference between CNN-predicted and manual segmentations of pelvic bones is provided in Additional file 3.

### SCORE results of pelvic bones

**Fig. 7:**
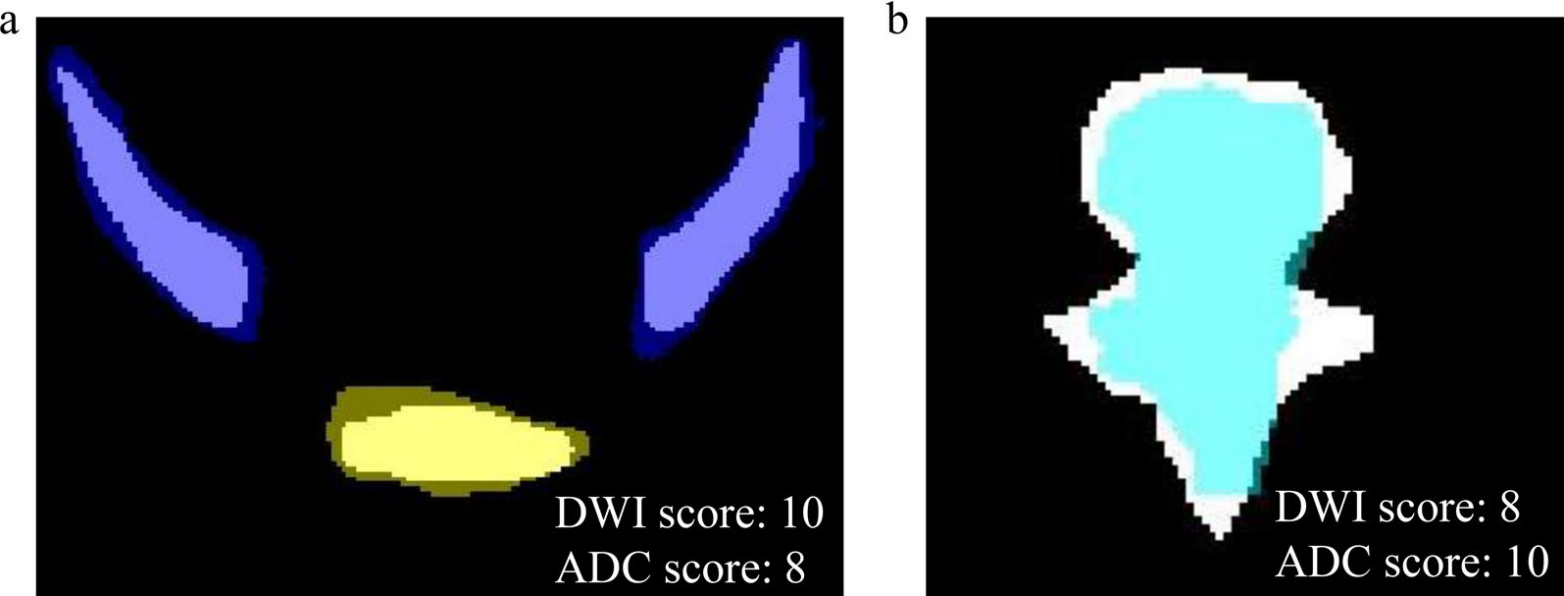
SCORE results on DWI and ADC images. **a** The range of the CNN-predicted segmentations of sacrococcyx (yellow label) and ilium (deep blue label) was slightly more extensive than the manual segmentations (white background), attaining DSC values of 0.92 and 0.88, respectively. According to the SCORE system, 10 on the DWI image and 8 on the ADC image were obtained. **b** The range of the CNN-predicted segmentation of lumbar vertebra (light blue label) was slightly smaller than manual segmentation (white background) with a DSC value of 0.82. According to the SCORE system, 8 on the DWI image and 10 on the ADC image were obtained

To identify if the qualitative segmentation results of the developed 3D U-Net model meet the requirement for clinical application, two readers scored every single CD on DWI and ADC images on testing and external validation sets (Fig. 7). A summary of the average scores at the patient level is provided in Table 5. The relatively high scores and excellent concordance between the two readers (ICC = 0.904, 95% confidence interval: 0.871–0.929) confirmed the feasibility of using the CNN clinically.

**Table 5:** The summary of patient scores on DWI and ADC images

Dataset	Sequences	Reader 1^#^			Reader 2^*^		
		Mean scores	t value	*p* value	Mean scores	t value	*p* value
Testing Set	DWI	7.94 ± 1.04	− 1.168	0.248	7.82 ± 1.10	− 1.587	0.118
	ADC	8.22 ± 0.78			8.20 ± 0.83		
External validation	DWI	8.03 ± 0.71	− 1.684	0.095	8.10 ± 0.69	− 1.291	0.199
	ADC	8.26 ± 0.77			8.23 ± 0.72		

Unless otherwise specified, data are mean ± standard deviations

^#^Reader 1 refers to a radiology expert with more than 15 years of reading experience

^*^Reader 2 refers to a radiology resident with 3 years of reading experience

## Discussion

In this study, a 3D U-Net model was trained to segment normal bony structures on pelvic DWI and ADC images to provide localisation information for subsequent detection of pelvic bone metastases. This method has been successfully used for segmentation of the lung lobes on Computed Tomography (CT) scans—a task that solves similar challenges related to the localisation of lung tumours [18]. In this research, the 3D U-Net model achieved good segmentation performance on pelvic bones with high average DSC values on testing and external validation sets. The quantitative volume comparison between CNN-predicted and manual segmentations was highly correlated and in close agreement.

Both quantitative and qualitative evaluations were done to determine the value of the 3D U-Net model for clinical applications. Generally, quantitative evaluation indicators are horizontally comparable among different technical studies [19–21]. However, the specified qualitative evaluation is more important when faced with different clinical problems raised in clinical research, which yields the difference between basic and clinical research in this field. Taking the evaluation of this semantic segmentation (multiple areas of pelvic bones) as an example, the quantitative evaluation indicators include DSC values and volume comparisons between CNN-predicted and manual segmentations. However, considering the different usage of DWI and ADC images on bone metastases evaluation, identical quantitative results on DWI and ADC images may result in different evaluation criteria and clinical acceptability [2, 22, 23]. Besides, on ADC maps, the DSC of lumbar vertebra in the testing set was significantly larger than in the external set (*t* = 2.564*, p* = 0.012), while the difference has no clinical significance since the DSC values were large enough (both with > 0.80).

Undefined performance metrics adapted to clinical requirements represent one of the barriers to the clinical evaluation and adoption of fully automated segmentation methods [24]. At present, the majority of qualitative evaluations of automated medical image segmentations are mainly based on visual observation [25–27], and the lack of standardised criteria on visual observations can introduce some degree of variability and inconsistency between intra- and inter-readers. It is therefore vital to ensure uniformity on the qualitative evaluation standard. In this study, a SCORE system was formulated to promote standardisation in the qualitative evaluation of pelvic bone segmentation on DWI and ADC images, potentially diminishing estimation variability and increasing precision.

DWI was primarily used to detect bone metastases. When the prediction range is slightly more extensive than the manual segmentation (i.e., A1) on DWI, it is still acceptable for lesion detection. The ADC map was mainly used to measure ADC values. The ROI used for the ADC value measurement should be contained within bone structures. Therefore, the slightly smaller range of CNN prediction than the manual segmentation (i.e., A2) can meet the demand of ADC value measurement.

Notably, these results present a trend that the ADC images outperformed the DWI images in terms of the average DSC value in both the testing (*P*_DWI vs ADC_ < 0.001) and external validation sets (*P*_DWI vs ADC_ < 0.001), while the SCORE results showed no significant difference between DWI images and ADC images (*p* > 0.05). We believe this is due to greater variation in signal intensity of DWI images than in ADC images among the different MR units, resulting in a lower DSC value. However, considering the clinical usage of DWI images for lesion localisation, the lower DSC value due to overestimation is not necessarily a flaw according to the SCORE system thus allowing for bias. The segmentation performance on DWI and ADC images acquired from vendor 1 was better than vendors 2 and 3 (DWI images: *P*_V1 vs V2 vs V3_ < 0.001; ADC images: *P*_V1 vs V2 vs V3_ < 0.001), which may be due to more data from vendor 1 were acquired in the clinical practice and collected during model development. These results remind us that multiple and multi-vendor data are necessary for segmentation algorithm development instead of single-vendor algorithms that are not suitable for real clinical applications.

Comprehensive clinical research based on deep learning is usually divided into multiple sequential steps, with each step employing deep learning or traditional image processing methods [28, 29]. Regarding the segmentation of kidney stones on CT images, the kidney is firstly segmented by the deep learning method, after which the high-density stone is identified using the traditional threshold segmentation method [30]. The division of the complex clinical tasks can not only improve the acceptability of the model but can also save training resources, which highlights the value of the clinicians participating in model training. This research focused on one of the sequential tasks to detect pelvic bone metastases using the deep learning method, aiming to achieve the localisation of pelvic bones. An algorithm for detecting bone metastases is to subsequently develop and, finally, achieve automation of both detection and localisation of pelvic bone metastases.

Substantial diversities across mpMRI images (different patients and vendors) make automated segmentation challenging for real clinical applications. In this study, a 3D U-Net model was supplied with MR data collected from three independent vendors with various parameters. The performance demonstrated the high robustness of the model to different technical parameters and scanner types, which would greatly benefit patients who undergo different scans in MRI examinations during routine clinical care. Moreover, to simulate clinical application scenarios, independent and consecutive MR datasets were collected from different periods as a source for external validation and to evaluate the generalizability of this CNN.

There are several limitations to this research. Like all supervised learning techniques, this method relies heavily on manual annotation which gives rise to user variability. No images with metastasis have been included in the dataset, and this CNN was not tested on pelvic bone structures containing metastatic lesions. Thus its performance in the segmentation of such cases still needs to be verified, and the generalisation of the model to the presence of these lesions need further confirmation. The SCORE system for qualitative evaluation of segmentation established by a radiology expert at our institution may be subjective and limited, and may necessitate a consensus from several experts from multiple institutions for actual clinical research. Furthermore, the 3D U-Net model was only trained and validated with retrospective data, while prospective evaluation still needs to be conducted, particularly on benign bone abnormalities and common imaging artifacts. Multicentre data may be required before the algorithm can be deployed in a clinical workflow.

## Conclusions

In conclusion, the presented 3D U-Net CNN can achieve automated and accurate segmentation of pelvic bones without metastases on DWI and ADC images acquired from different MR vendors. This work presents a promising step toward a highly desired automated mpMRI-based imaging methodology to detect skeletal metastases.

## Supplementary Information

**Additional file 1.** The network architecture of 3D U-Net.

**Additional file 2.** The Demographics of patients among different vendors.

**Additional file 3.** The percent of volume difference between CNN-predicted and manual segmentations of pelvic bones on DWI and ADC images.

## Abbreviations

3D: Three-dimensional
ADC: Apparent diffusion coefficient
CD: Connected domain
CT: Computed tomography
DSC: Dice similarity coefficient
DWI: Diffusion-weighted imaging
F-PSA: Free prostate-specific antigen
ICC: Intraclass correlation coefficient
LoA: Limits of agreement
mpMRI: Multiparametric magnetic resonance imaging
PSA: Prostate-specific antigen
T-PSA: Total prostate-specific antigen
WB-MRI: Whole-body MRI
