# Supplementary material for: Fully automated pelvic bone segmentation in multiparameteric MRI using a 3D convolutional neural network
Source: Insights Imaging. 2021 Jul 7;12:93. doi: 10.1186/s13244-021-01044-z (PMC8263843; doi:10.1186/s13244-021-01044-z)
Supplement: Supplementary file 1 — Additional file 1. The network architecture of 3D U-Net. [file 13244_2021_1044_MOESM1_ESM.docx]

***Additional file 1:***

**The network architecture of 3D U-Net**

The network architecture of 3D U-Net was illustrated in Figure S1. The 3D U-Net extended the U-Net architecture from Ronneberger et al [1] by replacing all 2D operations with their 3D counterparts [2]. It has an analysis and a synthesis path. In the analysis path, each layer contains two 3×3×3 convolutions, each convolution followed by a batch normalization (BN), a rectified linear unit (ReLU), and then a 2×2×2 maximum pooling with strides of two in each dimension. In the synthesis path, each layer consists of an upconvolution of 2×2×2 by strides of two in each dimension, followed by two 3×3×3 convolutions each followed by a BN and a ReLu. Each layer in the synthesis path has an up-convolution, which is concatenated with the correspondingly feature map from the analysis path. In the last layer a 1×1×1 convolution with the sigmoid activation function was used to reduce the number of output channels to the number of labels.


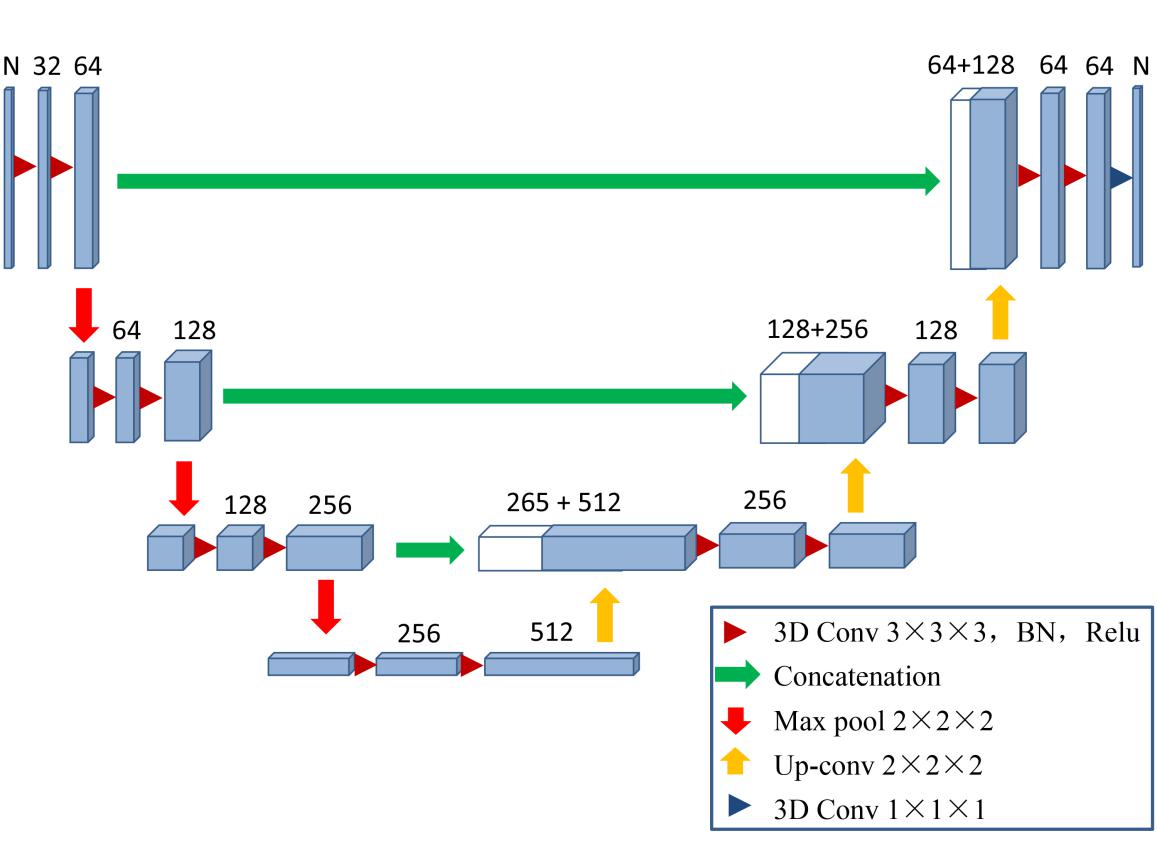


**Figure S1.** Network architecture of the three-dimensional (3D) U-Net [2].

The blue boxes are the feature maps of 3D MRI images. The numbers above the blue boxes represent the number of channels in each feature map. N: number of labels; Conv: convolution; ReLu: rectified linear unit; BN: batch normalization; Up-conv upconvolution.

**References:**

1. Ronneberger O, Fischer P, Brox T (2015) U-Net: Convolutional Networks for Biomedical Image Segmentation. In: International Conference on Medical image computing and computer‑assisted intervention. Springer 234-241

2. Çiçek Ö, Abdulkadir A, Lienkamp SS, Brox T, Ronneberger O (2016) 3D U-Net: learning dense volumetric segmentation from sparse annotation. In: International Conference on Medical image computing and computer‑assisted intervention Springer 424-432
