# Supplementary material for: Fully automated pelvic bone segmentation in multiparameteric MRI using a 3D convolutional neural network
Source: Insights Imaging. 2021 Jul 7;12:93. doi: 10.1186/s13244-021-01044-z (PMC8263843; doi:10.1186/s13244-021-01044-z)
Supplement: Supplementary file 2 — Additional file 2. The Demographics of patients among different vendors. [file 13244_2021_1044_MOESM2_ESM.docx]

***Additional file 2:***

**Table S1.** The Demographics of patients among different vendors

| Characteristic | Algorithm development^#^  (N = 264) | | | | | External Validation^*^  (N = 60) | | | | |
| --- | --- | --- | --- | --- | --- | --- | --- | --- | --- | --- |
|  | 3.0 T Discovery | 1.5 T Intera | 1.5 T Avanto | Statistical value | P value | 3.0 T Discovery | 1.5 T Intera | 1.5 T Avanto | Statistical value | P value |
| No. of patients | 197 | 47 | 20 | - | - | 41 | 11 | 8 | - | - |
| Age  (mean, y)  (SD) | 67.20  (9.51) | 67.04  (10.20) | 66.65  (10.67) | 0.662 | 0.718 | 65.12  (12.89) | 68.27  (13.30) | 62.75  (14.71) | 1.322 | 0.516 |
| PSA (median, ng/ml） | | | | | | | | | | |
| T-PSA  （range） | 10.01  (0.15,156) | 8.52  (0.77,76.78) | 10.87  (2.42,40.22) | 2.018 | 0.365 | 6.94  (1.38, 225) | 15.02  (4.49, 47.19) | 12.83  (7.10, 39.62) | 4.963 | 0.084 |
| F-PSA  （range） | 1.29  (0.09,14.37) | 1.15  (0.19,23.46) | 1.27  (0.39,3.77) | 0.194 | 0.907 | 1.36  (0.43,13.2) | 1.95  (0.33, 5.54) | 1.68  (0.86, 8.58) | 1.169 | 0.557 |
| F/T-PSA  （range） | 0.13  (0.02,0.36) | 0.15  (0.05, 0.35) | 0.12  (0.05,85.00) | 3.548 | 0.170 | 0.16  (0.06,0.65) | 0.12  (0.05, 0.13) | 0.17  (0.08, 0.22) | 5.641 | 0.060 |

SD: standard deviation; PSA: prostate specific antigen; T-PSA: total PSA; F-PSA: free PSA.

^#^ The data in algorithm development were collected between August 2018 and August 2019

^*^ The data in external validation were collected between January 2020 and March 2020
