# Supplementary material for: Fully automated pelvic bone segmentation in multiparameteric MRI using a 3D convolutional neural network
Source: Insights Imaging. 2021 Jul 7;12:93. doi: 10.1186/s13244-021-01044-z (PMC8263843; doi:10.1186/s13244-021-01044-z)
Supplement: Supplementary file 3 — Additional file 3. The percent of volume difference between CNN-predicted and manual segmentations of pelvic bones on DWI and ADC images. [file 13244_2021_1044_MOESM3_ESM.docx]

***Additional file 3:***

The accuracy of bone segmentation was also quantified by the volume calculation. The percent of volume difference were evaluated by taking the absolute mean difference between automated segmentation and manual annotation divided by the mean volume of manual annotations: $V\%=\left( \frac{\left| V_{P}-V_{M} \right|}{V_{A}} \right)\times100\%, ($V_P_ is the volume of predicted segmentation, V_M_ is the volume of manual segmentation, and V_A_ is the mean volume of manual annotations).

**Table S2.** The percent of volume difference between CNN-predicted and manual pelvic bones on DWI and ADC images

| Pelvic bones | DWI images | | | | ADC images | | | |
| --- | --- | --- | --- | --- | --- | --- | --- | --- |
|  | Testing Set  (N = 28,%) | External validation  (N = 60, %) | *U value* | *P value* | Testing Set  (N = 28, %) | External validation  (N = 60, %) | *U value* | *P value* |
| Lumbar vertebra | 17.32  (9.06, 27.68) | 12.17  (5.11, 22.85 ) | 664 | 0.115 | 10.97  (2.77, 25.29) | 13.6  (4.05, 22.67) | 2.564 | 0.012 |
| Sacrococcyx | 6.65  (3.81, 15.20) | 9.58  (4.33, 18.02) | 716 | 0.267 | 7.14  (3.04, 15.92) | 9.64  (6.02, 16.85) | 697 | 0.200 |
| Ilium | 7.31  (3.57, 11.47) | 8.62  (3.69, 14.63) | 740 | 0.370 | 5.12  (2.45, 8.05) | 6.02  (3.40, 9.75) | 742 | 0.380 |
| Acetabulum | 11.10  (7.62, 14.5) | 8.41  (4.71, 16.91) | 746 | 0.400 | 9.20  (5.86, 17.22) | 6.00  (2.81, 15.13) | 682 | 0.157 |
| Femoral head | 8.32  (3.48, 18.49) | 14.03  (4.77, 23.79) | 685 | 0.165 | 7.97  (3.08, 14.34) | 13.55  (5.39, 26.26) | 636 | 0.068 |
| Femoral neck | 5.98  (3.20, 11.33) | 5.88  (2.65, 12.89) | 818 | 0.844 | 4.58  (1.89, 8.86) | 6.94  (2.81, 9.73) | 687 | 0.170 |
| Ischium | 9.68  (4.95, 16.87) | 14.92  (6.94, 23.53) | 662 | 0.111 | 9.20  (4.20, 14.74) | 10.35  (5.01, 15.45) | 791 | 0.661 |
| Pubis | 17.36  (9.31, 25.60) | 17.19  (7.35, 30.10) | 814 | 0.816 | 10.17  (4.45, 19.02) | 10.26  (5.08, 24.62) | 824 | 0.886 |
| Average^#^ | 3.31  (1,12, 6.07) | 5.60  (2.31, 9.32) | 580 | 0.020 | 3.32  (1.14, 4.77) | 4.12  (1.71, 5.90) | 659 | 0.105 |

Data are median percentage, with the 25th and 75th percentages in parentheses.

Mann-Whitney U was used to compare the volume difference between testing set and external validation set.
